# Applications of Hydrogel and Aerogel Absorbent Pads in Food Packaging: From Exudate Management to Active and Intelligent Preservation

**DOI:** 10.3390/gels12090754

**Published:** 2026-08-23

**Authors:** Ke Zhang, Zhihua Li, Xiaowei Huang, Zhou Qin, Xiaodong Zhai, Junjun Zhang, Jiyong Shi

**Affiliations:** Agricultural Product Processing and Storage Lab, School of Food and Biological Engineering, Jiangsu University, Zhenjiang 212013, China

**Keywords:** gel-based materials, liquid absorption, active packaging, shelf-life extension

## Abstract

Absorbent pads are important materials for regulating exudate and local microenvironments in the packaging of high-moisture perishable foods. However, conventional absorbent pads often suffer from limited functionality, insufficient liquid retention, and a lack of active responsiveness. Hydrogels and aerogels, with tunable three-dimensional polymer networks, provide an important material basis for the design of new functional absorbent pads. This review focuses on the relationships among structure, function, and application, and compares hydrogels and aerogels in terms of network composition, crosslinking strategies, water absorption and retention mechanisms, and active compound loading and release behaviors. Structural design strategies, including multilayer structures, Janus structures, gradient pore structures, and micro/nano-reinforcement, are also summarized. On this basis, recent applications of hydrogel- and aerogel-based absorbent pads in the packaging of meat, aquatic products, fruits, vegetables, and edible fungi are discussed. Finally, the key challenges facing gel-based absorbent pads are analyzed, including adaptation to real food systems, release regulation, food-contact safety, and industrial-scale production. This review establishes a structure–function–application framework for gel-based absorbent pads and offers insights for designing sustainable active and intelligent food packaging.

## 1. Introduction

Food loss and waste have become critical issues in global food security, resource utilization, and environmental sustainability [1,2]. According to statistics from the Food and Agriculture Organization of the United Nations (FAO) and the United Nations Environment Programme (UNEP), approximately 13.2% of food worldwide is lost along the supply chain from post-harvest to pre-retail stages, while another 19% of food available to consumers is wasted at the retail, food service, and household levels. Among these perishable foods, meat, aquatic products, and fruits are particularly prone to exudate accumulation, microbial proliferation, lipid oxidation, and mechanical damage during refrigerated storage, transportation, and retailing because of their high moisture content, rich nutrient composition, or fragile tissue structure [3,4,5]. Related studies have shown that packaging for perishable foods should not only regulate moisture migration and the local microenvironment but also ensure microbial safety, maintain product quality, and extend shelf life [6,7]. In this context, sustainable practices in packaging design and life-cycle management have received increasing attention, including proper handling and disposal of packaging wastes to minimize environmental and health risks [8]. Therefore, the development of green packaging materials that can simultaneously improve food quality, safety, and shelf life has become an important focus in food science, materials science, and sustainable packaging [9].

Absorbent pads are commonly used auxiliary materials in the packaging of high-moisture perishable foods. They are mainly designed to absorb meat juice, blood exudate, condensed water, or juice released from damaged tissues, thereby reducing the accumulation of free liquid inside packages and improving product appearance [10,11]. Conventional absorbent pads are typically composed of cellulose, nonwoven fabrics, superabsorbent polymers, or plastic composite structures. Although they possess a certain liquid absorption capacity, they often suffer from poor biodegradability, limited functionality, insufficient liquid retention, risk of liquid re-exudation, and a lack of active preservation and intelligent response capabilities [11]. In recent years, advances in active packaging films and intelligent monitoring technologies have shown that food packaging materials are gradually shifting from passive barriers toward multifunctional systems integrating antibacterial and antioxidant functions, quality monitoring, and information feedback [12,13]. Therefore, absorbent pads are no longer merely liquid-absorbing materials, but are increasingly being developed as functional platforms that integrate exudate management, active compound loading, controlled release, and quality monitoring.

Hydrogels and aerogels provide an important material basis for the design of novel absorbent pads. Both are centered on three-dimensional polymer networks and can be constructed from bio-based materials such as sodium alginate, chitosan, pectin, starch, cellulose, gelatin, and proteins, offering good structural tunability and functional expandability. Progress in polysaccharide-based food packaging films and coatings has also demonstrated the broad application potential of natural polymers in sustainable food packaging [14,15,16,17]. Hydrogels are hydrated three-dimensional network materials with flexibility, swelling ability, and environmental responsiveness, making them suitable for absorbing and retaining food exudates. They can also regulate the release of active compounds in response to changes in pH, ionic strength, enzymes, or microbial metabolites [18]. Aerogels are generally obtained by drying wet gels and are characterized by low density, high porosity, large specific surface area, and open-pore structures, making them more suitable for rapid liquid uptake, liquid storage, cushioning protection, and efficient loading of antibacterial agents, antioxidants, or indicators [19].

Previous studies have reported the applications of hydrogels or aerogels in food packaging, active preservation, intelligent indication, and bio-based materials. For example, hydrogel systems have been used to absorb food exudates, immobilize natural pigments, and construct pH-responsive indicator labels, whereas aerogel systems have been applied for moisture absorption, loading of essential oils or polyphenols, delaying meat spoilage, and cushioning fragile fruits [20]. However, existing reviews have rarely provided a systematic comparison of the intrinsic relationship, structural differences, and functional complementarity between hydrogels and aerogels when they are used as gel-based absorbent pads. Against this background, this review summarizes the applications of hydrogel and aerogel absorbent pads in food packaging, with emphasis on their structural basis, crosslinking strategies, functional differences, and structural innovations. Their applications in the packaging of animal-derived foods, fruits, vegetables, and edible fungi are further discussed, along with key challenges related to real food validation, the balance between water absorption and quality preservation, responsive release, and industrial-scale production. This review establishes a structure–function–application framework for gel-based absorbent pads by systematically comparing hydrogels and aerogels from a unified perspective, and provides guidance for the design of sustainable active and intelligent food packaging.

## 2. Structural Basis and Relationship Between Hydrogels and Aerogels

Hydrogels and aerogels are two typical gel systems formed under different hydration states and structural preservation modes. Both are based on three-dimensional polymer networks and can generally be constructed from polysaccharides, proteins, cellulose derivatives, and their composite systems, thereby offering good structural designability and functional expandability in food packaging [21,22]. Understanding their structural basis and intrinsic relationship is a prerequisite for further comparing their functional differences in food absorbent pads.

### 2.1. Hydrogels

Hydrogels are hydrated three-dimensional network materials formed by the physical or chemical crosslinking of hydrophilic polymer chains. Early classical studies on hydrophilic gels have demonstrated that such materials can maintain stable polymer network structures under highly hydrated conditions [23]. Within the hydrogel network, a large amount of water can either interact with hydrophilic groups such as hydroxyl, carboxyl, amino, and amide groups around polymer chains or be confined within gel pores and crosslinked networks, thereby endowing hydrogels with strong hydration, swelling, and liquid-retention capacities [24]. Through appropriate crosslinking strategies such as ionic crosslinking or freeze–thaw-induced physical crosslinking, these polymer chains can be stabilized into three-dimensional networks with tunable swelling and retention properties [25]. Alginate and low-methoxyl pectin can form ionically crosslinked networks with Ca^2+^. Among them, alginate–Ca^2+^ gels are commonly interpreted using the egg-box model, whereas pectin–Ca^2+^ gelation behavior is strongly influenced by the degree of methoxylation, Ca^2+^ concentration, and gelation conditions [26]. In food packaging absorbent pads, the value of hydrogels mainly lies in their flexible swelling networks, which can absorb liquids released from meat, aquatic products, fruits, and vegetables. Meanwhile, hydrophilic groups and crosslinked structures within the network can immobilize liquids to a certain extent, thereby reducing the accumulation of free water inside packages and lowering the risk of liquid re-exudation under pressure. Previous studies have employed PVA, agarose, alginate, and other hydrogel systems for the design of food absorbent pads. For instance, He et al. [27] constructed a meat packaging absorbent pad using PVA/agarose as the matrix, demonstrating the combination of the flexible PVA network and the supporting role of agarose gel. Similarly, Sun et al. [28] prepared hydrogel absorbent pads based mainly on sodium alginate for chilled pork packaging, indicating that ionically crosslinked polysaccharide networks can serve as a structural basis for food exudate management materials.

However, the application of hydrogels in food absorbent pads also has certain limitations. Owing to their high intrinsic water content, hydrogels may face challenges related to microbial stability, reduced wet-state mechanical strength, and premature diffusion of active compounds during long-term storage. In addition, after absorbing large amounts of exudate, hydrogels may undergo excessive swelling, resulting in structural deformation, surface adhesion, or pressure-induced re-exudation, which can compromise packaging performance. Therefore, in absorbent pad design, it is necessary to balance the water absorption capacity, liquid-retention capacity, and structural stability of hydrogels by regulating crosslinking density, network composition, composite reinforcement, and surface structure.

### 2.2. Aerogels

Aerogels are generally dry porous materials obtained from wet gels through specific drying processes. During this process, the liquid within the gel network is replaced by air or gas, while the three-dimensional skeleton is retained as much as possible [29,30]. Early classical studies on aerogels by Kistler demonstrated that lightweight materials with a continuous solid skeleton and highly porous structure can be obtained by appropriately removing the liquid phase from gels [31]. Depending on the drying method, the products of wet gels are categorized as aerogels, cryogels, or xerogels. Strictly speaking, true aerogels with well-preserved nanoporous skeletons are primarily obtained via supercritical drying. Freeze-drying produces cryogels with micrometer-scale pores via ice-templating, which are often loosely referred to as aerogels in food-related literature. Ambient-pressure drying, however, usually leads to dense xerogels due to capillary collapse, unless the gel skeleton is sufficiently reinforced. Compared with hydrogels, the most prominent structural features of aerogels are their low density, high porosity, large specific surface area, and open-pore structure, which are also key characteristics distinguishing them from ordinary dried gels and dense film materials.

For food packaging absorbent pads, aerogels usually enter the packaging system in a dry state. When they come into contact with exudates from meat or aquatic products, liquids can rapidly penetrate into the aerogel interior under the effects of capillary action, pore adsorption, and hydrophilic groups. Therefore, aerogels are more suitable for rapid liquid uptake and liquid storage. Unlike hydrogels, which absorb water mainly through network swelling, the liquid absorption behavior of aerogels depends more strongly on pore size distribution, pore interconnectivity, surface wettability, and skeleton stability. For example, Chang et al. [32] constructed an aerogel absorbent pad from plasma-treated citrus pectin and chitosan for chilled pork packaging, demonstrating that regulation of the pore structure and surface functional groups of polysaccharide aerogels can influence their liquid absorption and preservation performance. The open porous structure not only facilitates the rapid penetration of exudates into the material but also provides considerable loading space for antibacterial agents, antioxidants, freshness indicators, or adsorptive components. Another important feature of aerogels is that their dry porous networks are favorable for the immobilization and protection of active substances. Active compounds can be distributed inside the pores or on the skeleton surface through adsorption, encapsulation, deposition, or composite incorporation, thereby reducing their rapid diffusion or loss before packaging use. Hosseini et al. [33] incorporated clove essential oil into a composite aerogel absorbent pad for chicken packaging, highlighting the potential of aerogel pore structures as carriers for volatile or hydrophobic active substances. After exudates enter the aerogel pores, the release of active substances can be triggered through multiple physicochemical pathways. These include dissolution-driven diffusion of water-soluble agents upon contact with moisture, matrix swelling that enlarges pore channels and accelerates mass transfer, pH-responsive dissociation of loaded complexes in response to acidic or alkaline spoilage metabolites, and enzymatic or hydrolytic degradation of the gel network induced by microbial enzymes or endogenous food proteases. For instance, Hou et al. [34] demonstrated that cinnamaldehyde release from aerogel pads was significantly accelerated under acidic conditions that mimic spoilage environments, exemplifying pH-triggered release coupled with exudate penetration. This feature gives aerogels great potential in active-preservation absorbent pads and intelligent responsive packaging.

Nevertheless, aerogels also have some limitations that should be considered. Because of their high porosity and lightweight skeleton, some aerogel materials may exhibit high brittleness, insufficient compression recovery, and structural collapse under pressure. Meanwhile, the drying methods used in aerogel preparation may affect cost, energy consumption, and scalability. Therefore, when aerogels are applied in food packaging absorbent pads, attention should be paid simultaneously to pore-structure stability, mechanical strength, wet-state integrity after liquid absorption, and the scalability of the preparation process.

### 2.3. Intrinsic Relationship Between Hydrogels and Aerogels

**Figure 1:**
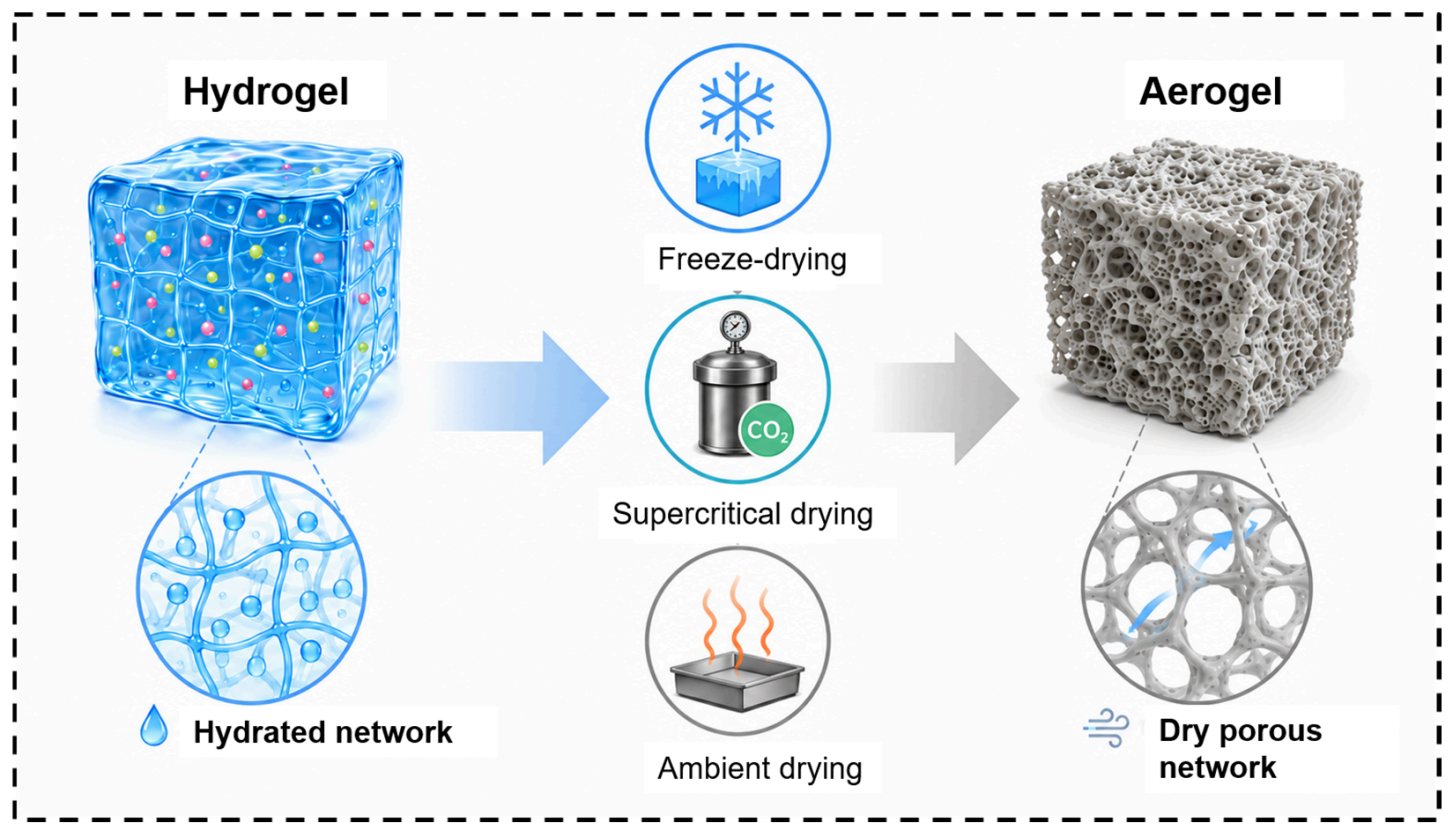
Structural relationship and drying routes from hydrogels to aerogels.

Hydrogels and aerogels should not be simply regarded as two completely separate classes of materials. Their common basis is a three-dimensional gel network, and their main difference lies in the dispersed phase filling the network pores. The pores of hydrogels are mainly filled with water; therefore, hydrogels are typically wet, soft, swellable, and capable of retaining liquids. In contrast, the pores of aerogels are mainly filled with gas, giving them dry, lightweight, porous, and rapid liquid-absorbing characteristics. As shown in Figure 1, many aerogels can be obtained from wet gels through freeze-drying, supercritical drying, or ambient-pressure drying. Groult et al. [35] compared the structures of hydrogels, aerogels, cryogels, and xerogels using pectin gel systems as an example, showing that the same gel precursor can form different material states through different drying routes. From a food packaging perspective, freeze-drying offers the most balanced trade-off between structural porosity, material safety, and practical scalability, whereas supercritical drying is less cost-effective and ambient-pressure drying risks pore collapse. However, this does not mean that all hydrogels can be converted into aerogels after drying. A wet gel can be transformed into an aerogel with low density, high porosity, and an open-pore structure only when the gel network has sufficient skeleton stability and the drying process can effectively avoid severe shrinkage and pore collapse. If a hydrogel undergoes obvious shrinkage or network collapse during conventional drying, it is more likely to form a xerogel, dried film, or dense porous material.

This relationship can be summarized as structural homology, different states, and functional complementarity. Structural homology means that both hydrogels and aerogels rely on polymer chains, crosslinking points, and three-dimensional network skeletons to maintain their material forms. Different states indicate that hydrogels are dominated by hydrated networks, whereas aerogels are dominated by dry porous networks. Functional complementarity means that the two materials have different but potentially synergistic functional roles in absorbent pads. Butylina et al. [36] compared PVA/cellulose nanocrystal hydrogels with their freeze-dried materials and showed that freeze-drying altered the morphology, crystallization behavior, and mechanical performance after rehydration. This indicates that the difference between hydrogels and dry porous materials is not merely the replacement of water in the pores by gas, but also involves the ability of the network skeleton to be preserved during drying and subsequent liquid uptake. Therefore, hydrogels are more suitable for providing flexible contact, wet-state liquid retention, and network confinement, whereas aerogels are more suitable for rapid liquid uptake, high-porosity loading, and pore-mediated mass transfer. In actual food packaging environments, hydrogels and aerogels may also show certain state-related transitions. After aerogels come into contact with exudates, their pores are gradually filled with liquid, and local regions may exhibit hydrated features similar to those of wet gels, but this does not mean that they return to the original hydrogel state. Conversely, wet gels can form aerogels only under appropriate drying conditions; otherwise, they are more likely to form xerogels or dense porous materials. Therefore, hydrogels and aerogels are not simply interchangeable, but are complementary systems sharing a common gel-network basis. This also suggests that crosslinking strategies are a key factor in understanding their formation processes, state differences, and functional performance.

## 3. Construction and Crosslinking Strategies of Gel Networks

The formation of gel-based absorbent pads relies on crosslinking interactions among polymer chains. Crosslinking strategies not only determine the stability of gel networks and their shape retention after liquid uptake, but also affect the preservation of pore structures in aerogel wet-gel precursors during drying. In general, hydrogels rely more on crosslinked networks to maintain wet-state stability after liquid absorption, whereas aerogels depend more strongly on crosslinked skeletons to preserve dry porous structures and structural integrity after liquid uptake.

### 3.1. Non-Covalent Physical Crosslinking

**Figure 2:**
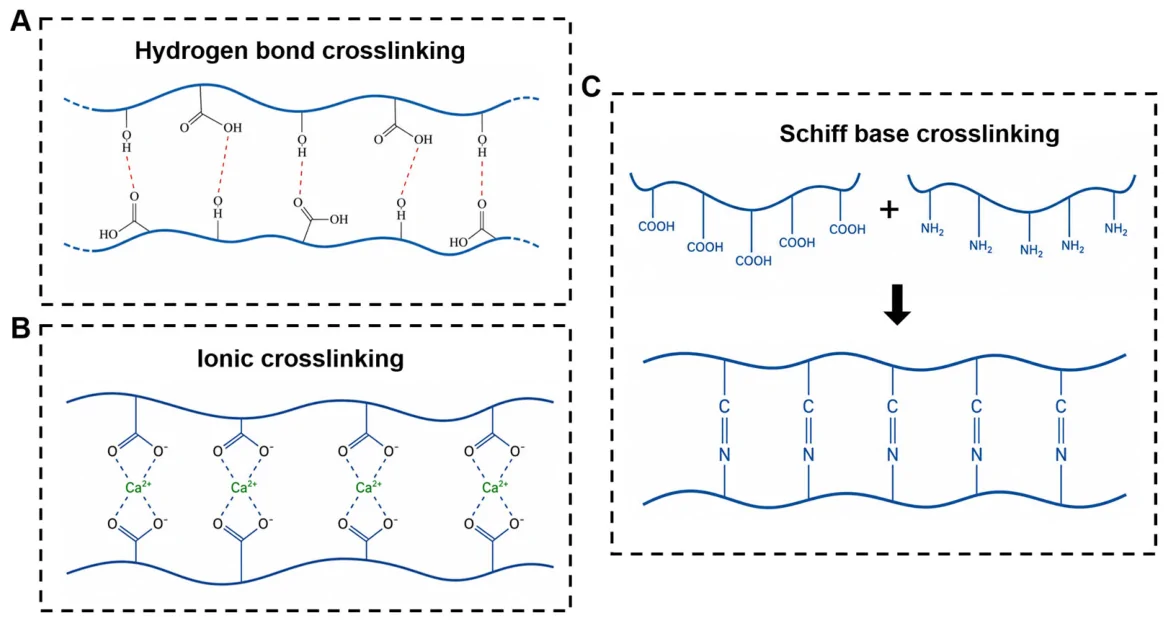
Schematic illustration of typical crosslinking strategies in gel absorbent pads: (**A**) non-covalent physical crosslinking; (**B**) ionic/coordination crosslinking; (**C**) covalent crosslinking.

Non-covalent physical crosslinking mainly relies on hydrogen bonding, electrostatic interactions, hydrophobic interactions, chain entanglement, and crystalline domains to form networks [37]. Polysaccharides, proteins, PVA, and cellulose derivatives contain functional groups such as hydroxyl, carboxyl, amino, and amide groups, which readily form gel structures through non-covalent interactions. Among them, hydrogen bonding is one of the most common physical crosslinking interactions in natural polymer gels. It can form dynamic connections between hydroxyl–hydroxyl and hydroxyl–carboxyl groups, thereby enhancing interchain interactions and stabilizing the gel network (Figure 2A). In addition, PVA can form microcrystalline domains through freeze–thaw cycles, which act as physical crosslinking points to maintain the stability of hydrogel networks [25]. For hydrogels, such crosslinking mainly restricts excessive chain extension, allowing the material to retain flexibility and a certain wet-state strength after swelling. For aerogels, its role is more closely related to stabilizing the wet-gel precursor, enabling the network to preserve a porous skeleton during freezing, solvent exchange, or drying [35]. Because non-covalent interactions are reversible, changes in water content, salt ions, proteins, and temperature may weaken network stability, leading to excessive swelling of hydrogels or skeleton softening of aerogels after liquid uptake. Therefore, non-covalent physical crosslinking is generally more suitable as a basic network-forming strategy and is often combined with ionic crosslinking, covalent crosslinking, or nano-reinforcement to improve the long-term stability of gel-based absorbent pads.

### 3.2. Ionic/Coordination Crosslinking

Ionic crosslinking is a common network-construction strategy in polysaccharide gels and mainly relies on electrostatic interactions or ionic bridging between charged groups and counterions [38]. Typical systems include sodium alginate–Ca^2+^, low-methoxyl pectin–Ca^2+^, and chitosan–tripolyphosphate (TPP). As shown in Figure 2B, polysaccharides such as alginate and low-methoxyl pectin can form ionically crosslinked networks via the well-established “egg-box” model. In this model, Ca^2+^ ions bind to the electronegative cavities formed between two antiparallel chains of guluronic acid blocks (G-blocks) in alginate or between de-esterified galacturonic acid regions in pectin. Each Ca^2+^ ion coordinates with multiple carboxyl and hydroxyl groups from adjacent chains, creating a series of junction zones that resemble an egg carton, which serve as physical crosslinking points to stabilize the three-dimensional network. In contrast, chitosan carries positively charged amino groups and thus forms polyelectrolyte complex networks with anionic crosslinkers such as TPP [26,39]. Coordination crosslinking places greater emphasis on coordination bonds between metal ions and ligand groups. Carboxyl, amino, hydroxyl, phenolic hydroxyl, and catechol groups in polysaccharides, proteins, and polyphenols can all serve as coordination sites to form coordination networks with Fe^3+^, Zn^2+^, Ca^2+^, and other metal ions. Compared with simple ionic crosslinking, coordination crosslinking generally exhibits stronger binding ability and a certain degree of dynamic reversibility, which helps enhance the stability of gel skeletons [40]. For hydrogel absorbent pads, ionic/coordination crosslinking facilitates rapid gel formation and helps maintain wet-state strength after liquid uptake. For aerogels, its role is more closely associated with stabilizing wet-gel precursors and helping preserve porous skeletons after drying. It should be noted that this type of crosslinking is often affected by metal ion diffusion, crosslinking gradients, and ion exchange, which may lead to structural heterogeneity between the interior and exterior of gels or local decrosslinking after liquid absorption [41]. Therefore, in the design of absorbent pads, attention should be paid to ion distribution, crosslinking uniformity, and the influence of competing ions in food systems.

### 3.3. Covalent Crosslinking

Covalent crosslinking connects polymer chains through chemical bonds and is generally more stable than physical or ionic crosslinking [42]. For hydrogels, covalent crosslinking helps improve wet-state strength and resistance to re-exudation after liquid uptake. For aerogels, it can reinforce the skeleton of wet-gel precursors, making them less prone to collapse during drying and subsequent liquid absorption. Therefore, covalent crosslinking is commonly used to enhance the structural stability of gel-based absorbent pads. Common covalent crosslinking strategies include citric acid crosslinking, genipin crosslinking, Schiff base reactions, and enzymatic crosslinking [43]. Citric acid can undergo esterification or amidation reactions with hydroxyl or amino groups in starch, cellulose, PVA, or chitosan, thereby improving the water resistance and mechanical stability of materials [44]. Genipin is commonly used in amino-containing polymer systems such as chitosan and gelatin and can serve as a natural crosslinking agent to improve the structural stability and functional performance of food packaging films [45]. As shown in Figure 2C, aldehyde groups in oxidized polysaccharides can react with amino-containing polymers such as chitosan or gelatin to form Schiff base networks [46]. It should be noted that stronger covalent crosslinking is not always preferable. Excessive crosslinking may reduce the swelling space and flexibility of gels, leading to a lower liquid absorption rate or increased brittleness of absorbent pads. Insufficient crosslinking, however, can result in low wet-state strength, poor liquid retention, or unstable pore structures in aerogels. Therefore, covalent crosslinking should be regarded as a strategy for regulating network stability rather than simply pursuing a higher crosslinking degree.

## 4. Structural and Functional Differences Between Hydrogels and Aerogels as Absorbent Pads

Although both hydrogels and aerogels are based on three-dimensional polymer networks, their functional performance in food absorbent pads is not identical. The fundamental reason lies in their differences in hydration state, pore structure, liquid uptake pathway, and active compound release route. Hydrogels are more similar to wet or swollen flexible networks, emphasizing liquid retention and responsiveness after water absorption. In contrast, aerogels are dry porous networks that emphasize rapid liquid uptake, pore-mediated liquid storage, and efficient loading. Therefore, in food packaging absorbent pads, hydrogels and aerogels are not simply interchangeable, but are suited to different functional requirements and structural design strategies.

### 4.1. Differences in Hydration State and Water Absorption/Retention Mechanisms

**Figure 3:**
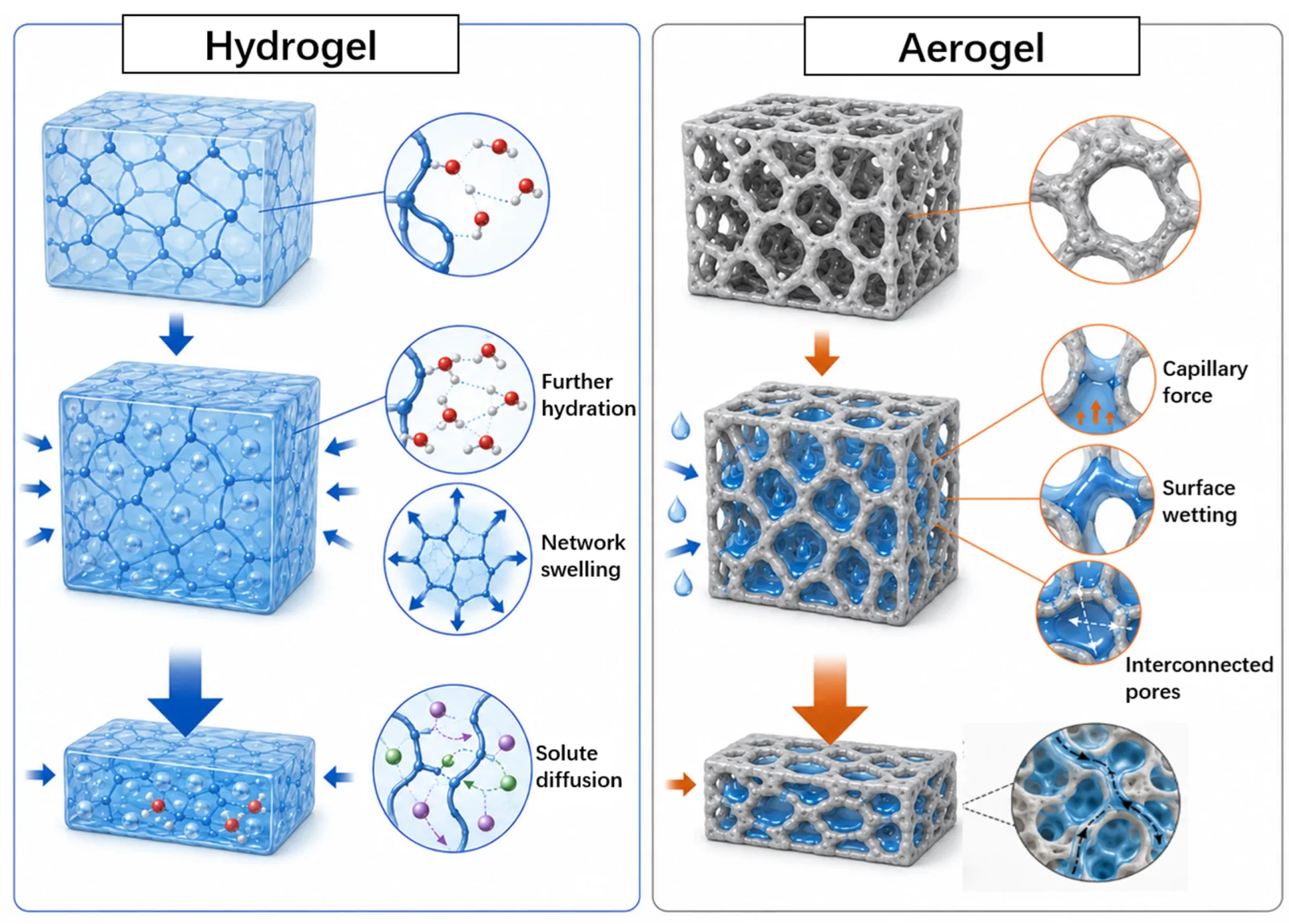
Differences in water absorption and retention mechanisms between hydrogels and aerogels.

As shown in Figure 3, hydration state is the basis for distinguishing the functional differences between hydrogels and aerogels as absorbent pads. Hydrogels usually exist in a hydrated or pre-swollen state, with a large amount of water already contained within their three-dimensional networks and polymer chains in a relatively extended and hydrated state. Therefore, when hydrogels come into contact with food exudates, liquid uptake is not simply a process of pore filling, but is accompanied by further hydration of hydrophilic groups, continuous network swelling, and solute diffusion within the gel phase. The PVA/agarose/anthocyanin hydrogel absorbent pad prepared by He et al. [27] for meat packaging exemplifies the integrated role of flexible hydrogel networks in exudate absorption, wet-state retention, and color indication. In such systems, hydrophilic groups such as hydroxyl, carboxyl, amino, and amide groups can form hydrogen bonds or electrostatic interactions with water molecules, allowing liquids to enter the gel network. Meanwhile, crosslinking points restrict unlimited extension of polymer chains, enabling the absorbed liquid to be confined within the three-dimensional network. Thus, hydrogels possess not only water absorption capacity but also a certain degree of liquid retention capacity. However, higher water absorption capacity is not always preferable. A lower crosslinking density facilitates network swelling, but may lead to decreased gel strength, structural deformation, or even rupture. In contrast, a higher crosslinking density improves wet-state stability but may restrict water entry into the network and reduce the swelling ratio.

Unlike hydrogels, aerogels typically enter food packaging systems in a dry state. Their pores are mainly filled with air, while the drying process preserves low density, high porosity, and open-pore structures. The pectin/chitosan aerogel absorbent pad prepared by Chang et al. [32] for chilled pork packaging demonstrates that the porous skeleton formed by drying polysaccharide wet gels can serve as a structural basis for rapid liquid uptake and exudate storage. After aerogels come into contact with exudates, liquids mainly enter the material under the driving forces of capillary action, surface wetting, and interconnected pore structures. Their liquid absorption behavior depends more on pore size distribution, pore interconnectivity, surface hydrophilicity, and skeleton integrity than on polymer network swelling alone. Therefore, aerogels are more favorable for rapidly capturing free exudates at the early stage of packaging and reducing liquid accumulation at the bottom of meat or aquatic products. In terms of liquid retention, aerogels mainly rely on pore-structure confinement, surface interactions, and compression-resistant skeleton stability. Hosseini et al. [33] used a bioactive aerogel as an absorbent pad for chicken packaging, demonstrating that aerogel pores can not only store exudates but also load active substances and participate in preservation. However, if the pores are too large or the skeleton is too brittle, the absorbed liquid may be released again under external pressure; if the pore structure is too dense, the liquid absorption rate may be reduced. Therefore, aerogel absorbent pads require not only high porosity but also appropriate pore size distribution, interconnected pore structures, and compression stability.

Overall, the differences in water absorption and retention between hydrogels and aerogels arise from their initial hydration states and network structures. Hydrogels emphasize network swelling, liquid confinement, and wet-state stability after liquid uptake, making them suitable for packaging environments requiring flexible contact and sustained liquid retention. Aerogels, in contrast, emphasize rapid liquid uptake, liquid storage, and active compound loading enabled by dry porous channels, making them suitable for scenarios requiring rapid removal of free exudates. Their functions in absorbent pads are not simply interchangeable but correspond to different requirements in absorption rate, liquid storage mode, and structural stability.

### 4.2. Differences in Active Compound Loading and Release

As absorbent pads evolve from single exudate-absorbing materials into platforms for active preservation and intelligent packaging, the loading and release capacity of active compounds has become an important criterion for evaluating hydrogels and aerogels. Active compound loading in hydrogels usually occurs during gel formation, in which antibacterial agents, antioxidants, natural pigments, nanoparticles, or enzyme-responsive components are embedded within wet networks [47]. Because hydrogels contain a large amount of water, hydrophilic active compounds can be readily dispersed within the network, and their release behavior can be regulated by crosslinking density, polymer chain interactions, and environmental responsiveness. The pH-responsive sodium alginate hydrogel prepared by Mao et al. [48], which delivered anthocyanins through a metal–polyphenol network, demonstrates that hydrogel networks can simultaneously serve as carriers for active compound immobilization, protection, and responsive release. Therefore, hydrogels are suitable for constructing sustained-release or stimuli-responsive absorbent pads [49].

The release process in hydrogels is generally associated with network swelling, diffusion, chain relaxation, and structural degradation. After an absorbent pad comes into contact with exudates, the gel network further swells, and active compounds diffuse outward under a concentration gradient. If the gel network is sensitive to pH, ionic strength, enzymes, or microbial metabolites, active compound release may also change along with the food spoilage process. The methylcellulose–sodium alginate hydrogel absorbent pad developed by Huang et al. [50], which incorporated anthocyanin-loaded metal–organic frameworks (anthocyanin@MOFs) for chilled pork preservation, reflects a strategy in which carrier particles and responsive networks synergistically regulate pigment immobilization and release. Such mechanisms are beneficial for constructing responsive-release absorbent pads that match food quality changes. However, active compound loading in hydrogels also has potential drawbacks. Because hydrogels are inherently wet, some active compounds may diffuse prematurely during storage or at the early stage of use, resulting in overly rapid release or increased migration risk. For natural pigments or small-molecule antibacterial agents, wet networks may also cause pigment leakage, color diffusion, or reduced active compound stability. Therefore, hydrogel absorbent pads should be designed to improve active compound immobilization and release stability by enhancing binding strength, introducing carrier particles, constructing double-network structures, or adopting multilayer isolation designs.

In contrast, active compound loading in aerogels relies more on dry-state adsorption, pore deposition, and surface immobilization. Owing to their large specific surface area and pore volume, active compounds can be distributed inside the pores or on the skeleton surface. Hou et al. [34] loaded a cinnamaldehyde-grafted chitosan complex into cellulose nanofiber/PVA aerogels for controlled-release packaging of fresh pork, demonstrating that the pore structure of aerogels can not only store active components but also be combined with pH-responsive complexes to regulate the release process. Compared with wet hydrogels, aerogels have certain advantages in loading volatile essential oils, hydrophobic antibacterial agents, antioxidants, and freshness indicators, as their dry environment helps reduce premature diffusion and leakage of some active compounds. The release of active compounds from aerogels is usually triggered by exudate penetration. When food exudates enter the aerogel pores, active compounds deposited or adsorbed within the pores gradually dissolve, diffuse, and are released into the local packaging environment. The thymol-nanoemulsion-loaded corn straw cellulose nanocrystal/acetylated starch aerogel prepared by Wang et al. [51] for chilled meat preservation demonstrates the advantages of porous aerogels in loading and sustained release of hydrophobic volatile active compounds. Rozykulyyeva et al. [52] further showed that the incorporation of essential oils into sponge-like aerogel absorbent pads not only imparted active functionality but also affected the structural durability of the materials.

Overall, hydrogels are more suitable for wet-state encapsulation and swelling–diffusion release, whereas aerogels are more suitable for dry-state adsorption and pore-triggered release. The former are advantageous because of their strong responsiveness, good flexibility, and release pathways that can be regulated through network structures. The latter are advantageous because of their large loading space, better initial stability, and release processes that can be coupled with exudate penetration. Therefore, future absorbent pad design should not simply compare which type of material has stronger release capacity, but should select appropriate loading and release strategies according to the properties of target active compounds, the generation pattern of food exudates, and preservation requirements [53].

## 5. Structural Innovations in Hydrogels and Aerogels

**Figure 4:**
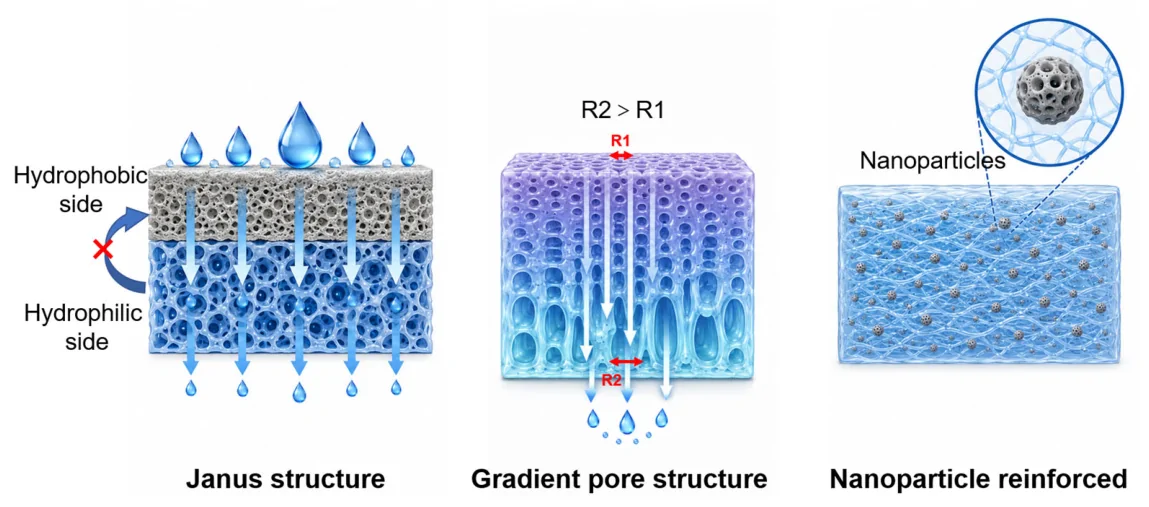
Structural innovation strategies for gel absorbent pads.

The performance of hydrogel and aerogel absorbent pads depends not only on material composition but also strongly on structural design. Conventional homogeneous absorbent pads usually integrate water absorption, liquid storage, antibacterial activity, antioxidant function, and indication capability within the same material layer. Although such designs are relatively simple to fabricate, they are prone to functional interference, premature release of active compounds, indicator migration, liquid re-exudation, and wet-state structural instability. Therefore, in recent years, the design of gel-based absorbent pads has gradually shifted from single homogeneous structures toward multilayer, Janus, gradient, and micro/nano-reinforced structures (Figure 4). Through spatial structural regulation, functions such as water transport, liquid storage, active release, and freshness monitoring can be separately designed in different regions, thereby improving the applicability of absorbent pads in real food packaging environments.

### 5.1. Multilayer and Janus Structural Design

The core of multilayer structural design lies in the spatial partitioning of functions such as food contact, water absorption and liquid storage, active release, and signal indication, allowing different functions to operate at appropriate positions [54]. For gel-based absorbent pads, the food-contact layer should ensure safety and suitable interfacial wettability while avoiding strong adhesion to the food surface or excessive dehydration. The water-absorbing and liquid-storage layer should possess high liquid uptake capacity and liquid retention ability to absorb and immobilize exudates released from meat, aquatic products, fruits, or vegetables. The active-release layer can be loaded with functional components such as antibacterial agents, antioxidants, or essential oils, whereas the indicator layer can immobilize natural pigments or other responsive probes to reflect changes in pH, volatile amines, or spoilage metabolites.

Compared with single-layer homogeneous absorbent pads, multilayer structures offer the advantage of reducing interference among different functions, as each layer can be independently optimized for its specific role [55]. However, they require careful control of interlayer adhesion, and increasing layer numbers may complicate fabrication and compromise structural integrity. When antibacterial agents, antioxidants, and indicators are simultaneously dispersed within the same gel network, the release of active compounds may affect the color stability of indicators, and indicators may migrate with exudates onto the food surface, raising concerns regarding safety and accuracy. Huang et al. [56] constructed a bilayer hydrogel active film involving red cabbage anthocyanins for pork preservation and freshness monitoring, demonstrating that multilayer hydrogel structures can integrate preservation and indication functions through functional partitioning. At the same time, this study also suggests that the location and migration behavior of indicator components such as anthocyanins require further control. For aerogel systems, multilayer studies directly targeting food absorbent pads remain limited, but related aerogel studies can provide useful references for structural design. Zhou et al. [57] fabricated a core–shell nanocellulose-based food packaging aerogel by coaxial 3D printing and confined silver nanoparticles (AgNPs) within the core region, indicating that aerogels can regulate active compound immobilization and release through spatial partitioning. Although the bilayer aerogel prepared by Qi et al. [58] was not designed for food packaging, its strategy of achieving functional partitioning through different aerogel layers also suggests that aerogels can be developed into layered structures. These findings indicate that multilayer structures are not simply about increasing the number of material layers, but about reducing mutual interference among water absorption, preservation, release, and indication functions through spatial partitioning.

In contrast to multilayer structures, Janus structures are defined not by the number of layers but by the significant physicochemical asymmetry between two sides of a material, which can be achieved either in a single layer (e.g., via surface modification, asymmetric crosslinking, or single-sided coating) or in a bilayer/multilayer configuration. The core design principle of Janus structures lies in creating directional differences in wettability, pore architecture, surface charge, or mass transfer pathways across the two sides, enabling functions such as unidirectional liquid transport and anti-backflow [59]. Shan et al. [59] prepared a gelatin hydrogel/ethyl cellulose bilayer film by combining a hydrophilic gelatin hydrogel layer with a hydrophobic ethyl cellulose layer for white mushroom preservation, demonstrating the role of hydrophilic/hydrophobic layered structures in humidity regulation and interfacial control. In this bilayer case, the Janus property arises from the asymmetry between the two layers, but Janus structures can also be realized in monolithic materials without multiple physical layers. Janus structures offer distinct advantages in directional liquid transport and anti-backflow, which are highly desirable for preventing re-exudation, yet they demand higher fabrication precision and face greater challenges in scalable production. The choice or combination of these strategies should be guided by specific packaging requirements and processing feasibility.

Overall, multilayer structures emphasize the spatial separation of food-contact safety, water absorption and liquid storage, active release, and signal readout, whereas Janus structures place greater emphasis on liquid transport direction and interfacial asymmetry. The two strategies are complementary and can be integrated—for instance, by embedding a Janus interface into a multilayer architecture to achieve unidirectional exudate uptake within a spatially partitioned pad. Future gel-based absorbent pads can be designed according to the logic of a food-contact regulation layer, a water-absorbing and liquid-storage layer, an active-release layer, and an indicator layer. By further integrating hydrophilic/hydrophobic differences or pore-structure gradients, exudates can rapidly enter the internal liquid-storage layer, while active compound release and color response can be controlled in locations that are safe, stable, and easy to observe.

### 5.2. Gradient Pore Structures and Anisotropic Structures

In addition to layered wettability differences, pore-structure gradients and anisotropy are also important strategies for improving the performance of gel-based absorbent pads. The pore structures of conventional homogeneous gel materials are usually relatively random, and liquids may diffuse disorderly within the material after entering, resulting in unstable liquid absorption rates, uneven local liquid storage, or pressure-induced re-exudation. By constructing gradient pore structures, oriented channels, or anisotropic networks, the migration pathway of liquids within absorbent pads can be deliberately regulated, enabling exudates to enter the internal liquid-storage region more rapidly while reducing liquid retention on the food-contact surface.

Gradient pore structures generally refer to materials with different pore sizes, pore densities, crosslinking densities, or component distributions in different regions. Studies on gradient hydrogels have shown that hydrogels are not necessarily homogeneous networks, and their structures, compositions, or functions can vary continuously or stepwise along spatial directions [60]. For example, Guo et al. (2021) [61] prepared gradient anisotropic cellulose hydrogels through CaCl_2_ solution diffusion, in which the orientation degree of cellulose chains gradually changed along the ion diffusion direction. This indicates that ion-diffusion crosslinking can be used to construct hydrogel networks with directional structural differences. For food absorbent pads, this concept can be translated into designs in which crosslinking density or pore structure gradually changes from the food-contact surface to the internal liquid-storage region, thereby balancing surface resistance to re-exudation with internal liquid-storage capacity.

Anisotropic structures emphasize direction-dependent mass transfer, swelling, or mechanical behavior of materials. For hydrogels, directional freezing is a commonly used method for constructing oriented pores. Zhang et al. [62] prepared PVA hydrogels with oriented pore structures and anisotropic mechanical properties through directional freezing–thawing. Barrow and Zhang [63] further fabricated oriented porous stimuli-responsive hydrogels using directional freezing and frozen UV polymerization, and demonstrated that the oriented pore morphology could be retained in the hydrated state while showing compression strength and diffusion behavior related to the freezing direction. Although these studies were not conducted for food packaging applications, they indicate that pore orientation in hydrogels can affect wet-state mass transfer and mechanical responses, providing a basis for the design of liquid introduction, diffusion control, and liquid-retention stability in absorbent pads.

For aerogels, gradient pore and anisotropic structures are usually more directly reflected in dry-state pore alignment and directional transport channels. The growth direction and rate of ice crystals during freezing can influence pore-wall arrangement, pore size distribution, and pore interconnectivity. Therefore, porous skeletons can be regulated through directional freezing, bidirectional freezing, or stepwise freezing. The anisotropic cellulose nanofiber/poly(vinyl alcohol)/graphene oxide (CNF/PVA/GO) aerogel prepared by Zhou et al. [64] exhibited low density, high porosity, and oriented pore structures, as well as good compression stability and adsorption capacity. Although this study focused on oil adsorption rather than food absorbent pads, it demonstrates that oriented channels and stable skeletons can help improve liquid entry efficiency and structural retention under pressure, offering useful insights for the design of aerogel absorbent pads.

In food packaging scenarios, gradient pore and anisotropic structures can also be combined with active compound release and indicator functions. Active components can be preferentially distributed along liquid-flow pathways or in internal liquid-storage regions, so that local release is triggered when exudates enter the material, thereby reducing ineffective loss at the early stage of packaging. Indicators can also be immobilized in specific regions to respond to exudates or volatile spoilage metabolites without directly contacting the food. Thus, gradient pore structures and anisotropic structures are not only strategies for optimizing water absorption capacity, but also important foundations for achieving directional release and precise indication.

### 5.3. Micro/Nano-Reinforcement and Interfacial Regulation

In real food packaging environments, hydrogel and aerogel absorbent pads need to withstand complex conditions such as transportation, stacking, exudate infiltration, and low-temperature storage. Therefore, relying solely on a single polysaccharide or protein network often makes it difficult to simultaneously achieve high liquid uptake, strong liquid retention, good mechanical stability, and functional loading. Micro/nano-reinforcement and interfacial regulation can improve the overall performance of gel-based absorbent pads by enhancing the network skeleton, pore structure, and surface interactions. Common reinforcing components include nanocellulose, clay, silica, metal or metal oxide nanoparticles, protein nanoparticles, polysaccharide nanoparticles, and metal–organic frameworks.

Nanocellulose is one of the most promising reinforcing components for gel-based absorbent pads. Its high aspect ratio, abundant hydroxyl groups, and good mechanical strength help construct stable hydrogen-bonding networks and improve the skeleton strength and compression resistance of hydrogels or aerogels [65,66]. Thomas et al. [67] summarized the renewability, mechanical reinforcement capability, and multifunctional application potential of nanocellulose, indicating that it is suitable as an important reinforcing platform for bio-based gel materials. In aerogel absorbent pads, nanocellulose can not only strengthen pore-wall structures but also improve pore interconnectivity and liquid retention. In hydrogels, nanocellulose can act as physical crosslinking points or a reinforcing phase, thereby improving wet-state strength and reducing structural damage caused by excessive swelling.

Interfacial regulation is equally important. Food exudates are complex systems containing proteins, salts, lipids, and microbial metabolites. The surface wettability, charge state, and functional group composition of gel-based absorbent pads directly affect liquid uptake rate, active compound binding, and microbial adhesion. Chang et al. [32] modified citrus pectin by plasma treatment to alter its functional group composition and hydration capacity, and then combined it with chitosan to construct an aerogel absorbent pad for chilled pork preservation. This study showed that interfacial functional group regulation can improve the water absorption and adsorption behaviors of aerogels and further influence preservation performance in real food systems. Micro/nano-components can not only reinforce the skeleton structure and interfacial interactions of gel-based absorbent pads but also impart antibacterial, antioxidant, adsorption, and indication functions. For example, nanocellulose, bacterial cellulose, graphene oxide, and clay can improve the mechanical strength and structural stability of gels, whereas silver nanoparticles, metal oxides, essential oil nanoemulsions, and metal–organic frameworks can further provide antibacterial, sustained-release, adsorption, or indication functions. Yang et al. [68] introduced carboxymethyl cellulose (CMC)-stabilized AgNPs into bacterial cellulose-/citric-acid-crosslinked aerogels to prepare CMC@AgNPs/bacterial cellulose/citric acid (CMC@AgNPs/BC/CA) antibacterial aerogel absorbent pads, demonstrating that micro/nano-components can synergistically improve the performance of gel-based absorbent pads through structural reinforcement and functionalization.

However, micro/nano-reinforcement does not mean that more fillers are always better. Excessive nanofillers may block pores, reduce liquid absorption rates, or lead to material embrittlement, reduced transparency, and processing difficulties. For food packaging applications, nanomaterial migration, food-contact safety, and regulatory compatibility must also be considered. Therefore, micro/nano-reinforcement should serve clear structural objectives, such as improving wet-state strength, stabilizing pore structures, regulating wettability, immobilizing active compounds, or improving release behavior, rather than simply adding functional fillers. Future structural innovation in gel-based absorbent pads should balance performance improvement, food safety, and industrial scalability.

## 6. Applications of Gel-Based Absorbent Pads in Food Packaging

**Figure 5:**
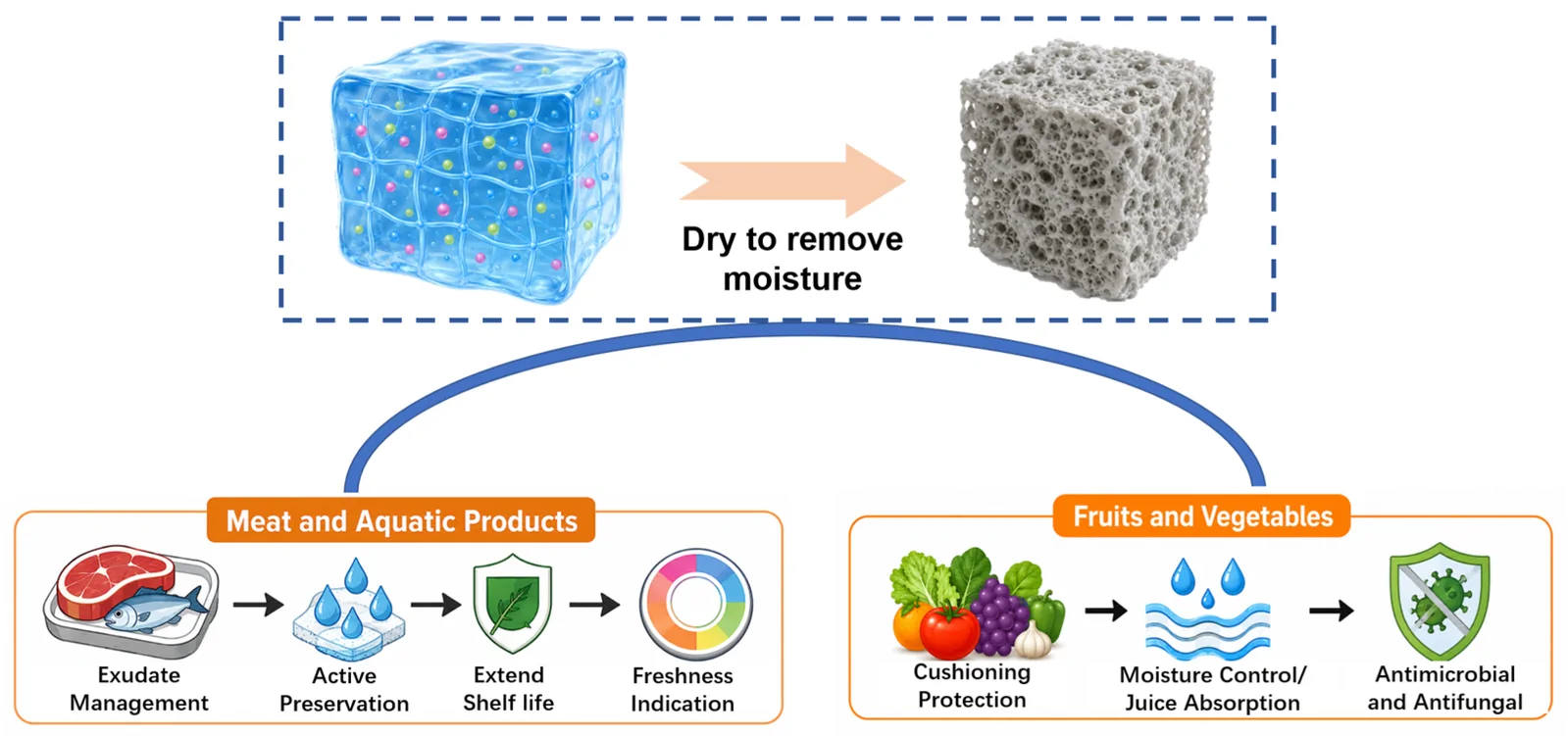
Application scenarios of gel-based absorbent pads in different food systems.

As illustrated in Figure 5, the applications of gel-based absorbent pads are mainly concentrated in two types of high-moisture perishable foods. One type includes meat and related animal-derived foods, for which the core issues are exudate accumulation, microbial proliferation, protein degradation, lipid oxidation, and freshness changes, thus requiring absorbent pads that can not only absorb liquids but also deliver antibacterial and antioxidant activities to retard spoilage. The other type includes fruits, especially fragile fruits such as berries, wax apples, and strawberries, where the major problems are mechanical damage, skin rupture, juice leakage, condensed water accumulation, and fungal contamination caused by compression and vibration during transportation, thus demanding cushioning protection, humidity/juice regulation, and antifungal functions. These distinct requirements, as highlighted in recent reviews on functional packaging materials [69], have inspired the development of tailored gel structures—from highly swellable hydrogels for meat juice management to lightweight, compressible aerogels for fruit cushioning—along with the integration of additional functionalities such as active compound release and freshness indication.

### 6.1. Applications in Meat and Aquatic Products

Meat and aquatic products are prone to exudate generation during refrigerated storage, transportation, and retailing. Exudates contain proteins, amino acids, salts, and other nutrients, which not only affect package appearance and consumer acceptance but also provide favorable conditions for microbial proliferation, protein degradation, lipid oxidation, and increases in total volatile basic nitrogen (TVB-N) [70]. Table 1 summarizes the material compositions, water absorption/swelling performance, and application effects of representative non-gel absorbent pads, hydrogels, and aerogel absorbent pads in meat and aquatic product packaging. It can be seen that related studies have gradually expanded from single drip-loss control to multifunctional directions involving exudate management, active preservation, and freshness indication.

Conventional absorbent pads mainly rely on cellulose, nonwoven fabrics, or superabsorbent polymers. Although they possess a certain liquid absorption capacity, their functions are usually limited. The study by Pettersen et al. [71] on chicken breast packaging showed that absorbent pads and packaging parameters can affect drip loss and quality performance, indicating that absorbent pads are not merely liquid-absorbing materials placed at the bottom of packages, but should be optimized according to food type and packaging conditions. Khunta et al. [72] applied poly(butylene adipate-co-terephthalate)/thermoplastic starch (PBAT/TPS) bio-based absorbent pads to pork packaging and evaluated color, total plate count (TPC), total volatile basic nitrogen (TVB-N), pH, and drip loss at 4 °C. The results showed that the absorbent pad with a PBAT/TPS ratio of 70:30 performed similarly to commercial plastic absorbent pads. Although this system is not a typical hydrogel or aerogel, it suggests that absorbent pad evaluation should be expanded from a single liquid absorption capacity to food quality, packaging applicability, and sustainability.

In gel-based systems, hydrogels place greater emphasis on swelling-based liquid uptake and network-mediated liquid retention. The sodium alginate/potassium-doped carbon dot hydrogel absorbent pad constructed by Sun et al. [28] for chilled pork preservation demonstrated the potential of hydrogel networks in exudate absorption, functional component immobilization, and active preservation. However, hydrogels may experience reduced wet-state strength, excessive swelling, or liquid re-exudation after absorbing liquids. Therefore, their practical application requires a balance among water absorption capacity, wet-state stability, and food-contact suitability. In contrast, aerogels enter packaging systems as dry porous structures and are more suitable for rapidly capturing exudates from meat or aquatic products, while storing liquids and loading active components through their pore structures. Yang et al. [68] introduced AgNPs formed by in situ reduction with CMC into bacterial cellulose-/citric-acid-crosslinked aerogels for fresh meat preservation, demonstrating that aerogel absorbent pads can synergistically achieve liquid uptake, liquid storage, and antibacterial effects through porous skeletons, nano-antibacterial components, and crosslinked structures.

Simply absorbing exudates is not sufficient to fully inhibit the spoilage of meat and aquatic products, because the nutrient-rich liquid absorbed inside absorbent pads may still become a microenvironment for microbial growth. Therefore, antibacterial, antioxidant, and controlled-release functions are often further incorporated into gel-based absorbent pads. Hou et al. [34] loaded a cinnamaldehyde-grafted chitosan complex into cellulose nanofiber/PVA aerogels. Release experiments showed that environmental changes increased the cinnamaldehyde release ratio from 10.3% to 68.4%, and the shelf life of fresh meat was extended by approximately 4 d. This indicates that liquid uptake by aerogel pores can be coupled with active compound release, making the preservation effect more consistent with the food deterioration process. Zhang et al. [73] developed sodium alginate/PVA/green tea extract (SA/PVA/GTE) aerogel pads for salmon preservation. The 3.0% GTE group exhibited a uniform three-dimensional interpenetrating network, a water absorption capacity of 2452.31%, and a hardness of 6308.61 g. In addition, hydrogen bonding between GTE and the matrix prolonged the diffusion pathway of antioxidants, extending the shelf life of salmon from 8 to 12 d. This example shows that, for aquatic product packaging, aerogels can not only absorb exudates but also serve as sustained-release carriers for natural active compounds, thereby integrating water absorption, antioxidant activity, antibacterial function, and biodegradable packaging performance.

In addition to active preservation, gel-based absorbent pads can also serve as platforms for monitoring meat freshness. During meat spoilage, proteins and nitrogen-containing compounds are decomposed by microorganisms and endogenous enzymes, producing ammonia, amines, and other volatile alkaline substances, which lead to increases in pH and TVB-N [76]. Therefore, intelligent meat packaging often relies on anthocyanins, curcumin, alizarin, betalains, or other pH- and amine-responsive indicators [77,78]. Zhang et al. [74] prepared sodium alginate/starch (SA/ST) aerogel pads co-loaded with black wolfberry anthocyanins and green tea extract, in which the porous network was used to absorb beef exudates, green tea polyphenols provided antibacterial and antioxidant effects, and copigmentation improved anthocyanin color stability. This system achieved a water absorption capacity of 2673%, a 2,2-diphenyl-1-picrylhydrazyl (DPPH) radical scavenging activity of 82.26%, and inhibition zones of 11.63 mm and 9.56 mm against *Escherichia coli* (*E. coli*) and *Staphylococcus aureus* (*S. aureus*), respectively. It also indicated the beef spoilage process through a visible color change from purple to blue. The juglone-loaded agarose hydrogel developed by Wang et al. [75] also showed rapid color development under low-concentration TVB-N gas and exhibited color responses consistent with changes in TVB-N and total viable count (TVC) in pork and fish. It should be noted that intelligent absorbent pads directly contact meat juice, and pigments may be affected by proteins, lipids, salts, and the color of meat exudates. Indicator migration and the representativeness of local responses also require further evaluation.

Overall, the development pathway of gel-based absorbent pads for meat and aquatic products is relatively clear. First, they improve package appearance and local microenvironments by absorbing and immobilizing exudates. Second, they delay microbial proliferation and oxidative spoilage through antibacterial, antioxidant, or controlled-release components. Finally, they enable freshness monitoring through pH- or volatile amine-responsive indicators. Hydrogels are more suitable for constructing flexible liquid-retention and responsive indication platforms, whereas aerogels are more suitable for building rapid liquid uptake, high-loading, and sustained-release preservation platforms. In the future, multilayer, Janus, or hydrogel/aerogel composite structures may be used to further integrate the advantages of both systems, enabling water absorption, preservation, and indication functions to be spatially coordinated rather than simply combined.

### 6.2. Applications in Fruits, Vegetables, and Edible Fungi

Unlike meat and aquatic products, the core issue in fruit packaging is not always exudate accumulation, but rather mechanical damage caused by vibration, compression, collision, and stacking during postharvest transportation and retailing. Fragile fruits such as berries, wax apples, strawberries, cherries, and peaches have thin skins and high moisture content, and even slight damage can lead to cell rupture, juice leakage, enhanced respiration, accelerated browning, and microbial invasion [79]. Table 2 summarizes representative applications of hydrogel and aerogel pads in systems such as wax apples, blueberries, strawberries, bananas, and straw mushrooms in recent years. Overall, aerogels are generally more suitable for cushioning protection and active preservation of fragile fruits, whereas hydrogels are relatively more suitable for local humidity regulation and quality maintenance in climacteric fruits and small edible fungi.

The study by Hou et al. [80] on chitosan-based aerogel cushioning packaging for wax apple preservation well illustrates this logic. In this study, chitosan/PVA aerogels were first optimized, followed by the construction of chitosan/montmorillonite aerogels, and then copper nanoparticle-loaded antibacterial fibers were further introduced to form aerogels. Under simulated transportation and storage conditions, the aerogel group reduced the decay rate by 66%, decreased weight loss by 5%, increased firmness by 6 N, reduced juice yield by 7%, decreased relative electrolyte leakage by 13%, and extended shelf life by 4 d at 20 °C compared with the control group. This case indicates that aerogel pads in fruit packaging should not be understood merely as materials for absorbing leaked juice, but rather as cushioning pads that integrate mechanical protection, local liquid absorption, and antibacterial preservation. Their functional pathway involves first reducing mechanical damage through cushioning, and then lowering the risk of secondary spoilage by absorbing local juice and inhibiting microbial growth.

Berries such as blueberries are also suitable for aerogel cushioning packaging. Wang et al. [81] developed chitosan-based CMC-Cu aerogel cushioning packaging, in which montmorillonite was used to load clove essential oil and nanocellulose was combined to immobilize copper nanoparticle-loaded antibacterial fibers, aiming to resist both mechanical damage and microbial contamination. This material achieved inhibition rates close to 100% against *E. coli* and *S. aureus* and extended the storage period of blueberries by approximately 3 d at 20 °C and 85% relative humidity. The study also showed that the immobilization effects of montmorillonite and nanocellulose could delay the release of antibacterial and antioxidant components, enabling the aerogel packaging to provide not only impact cushioning but also sustained preservation. This follows the same logic as the wax apple case: the advantage of aerogel pads for fruit packaging does not lie in antibacterial activity or water absorption alone, but in the combined effects of mechanical protection, moisture/juice regulation, and active preservation.

For fruits with a high risk of decay, such as strawberries, gel pads play a more important role in antifungal protection, humidity control, and sustained release of active compounds. Zhou et al. [82] prepared dialdehyde holocellulose nanofiber emulsion aerogel pads for strawberry preservation, in which dialdehyde holocellulose nanofiber-stabilized emulsions served as an effective approach for constructing antibacterial aerogel absorbent pads, allowing natural active compounds to be distributed within the porous network and exert sustained effects. Meng et al. [83] further constructed bio-aerogels from bamboo waste for strawberry preservation, demonstrating that agricultural waste can also be converted into low-cost and sustainable aerogel materials for fruit packaging. Compared with the wax apple and blueberry cases, the strawberry-related studies place greater emphasis on fungal control under high-humidity conditions and the sustainability of packaging pads, suggesting that aerogel applications in different fruits are not limited to cushioning protection but can also extend to active antifungal preservation and green packaging.

Moisture regulation in fruit packaging also differs from that in meat packaging. Intact fruits mainly face transpiration-related water loss, respiratory moisture, condensed water inside packages, and local juice leakage after damage, whereas fresh-cut fruits and small edible fungi are more prone to water loss, tissue softening, and microbial contamination [85,86]. Therefore, gel pads used for fruit and vegetable packaging need to balance the absorption of excess moisture with the prevention of excessive dehydration. Insufficient water absorption may allow condensed water and juice to promote fungal growth, whereas excessive water absorption may aggravate water loss and wilting, affecting firmness, color, and sensory quality. The open-pore structure of aerogels can absorb local juice and condensed water while reducing mechanical impact through an elastic porous skeleton. In contrast, hydrogels are more suitable for local moisturizing, active compound loading, and sustained release in fresh-cut fruits, climacteric fruits, or small edible fungi. Gang et al. [84] constructed a carboxymethyl cellulose/PVA/pyrazole acid composite hydrogel packaging system for postharvest banana preservation, indicating that hydrogel networks can serve as biodegradable active packaging matrices to delay quality deterioration in climacteric fruits through moisture regulation and active compound release. Jin et al. [87] prepared CMC-PVA-ethylene glycol diglycidyl ether (EGDE) hydrogels for the postharvest preservation of small packaged straw mushrooms, further showing that hydrogels can maintain a suitable moisture environment inside packages through hydrophilic networks and crosslinked structures, thereby helping preserve the overall postharvest quality of small fruits, vegetables, or edible fungi. Unlike aerogels, which mainly provide cushioning and liquid absorption, hydrogels in fruit and vegetable packaging are more advantageous in maintaining local humidity, loading active compounds, and regulating release processes.

Overall, the development focus of gel-based pads in fruits, vegetables, and edible fungi differs from that in meat and aquatic products. Meat and aquatic products are more suitable for integrated absorbent pads combining water absorption, antibacterial/antioxidant activity, and freshness indication, whereas fruits, vegetables, and edible fungi are more suitable for protective pads integrating cushioning, humidity/juice regulation, and antibacterial/antifungal functions. Aerogels show more obvious advantages in the packaging of fragile fruits, mainly because of their lightweight, porous, compressible, and easily functionalized structures. Hydrogels, on the other hand, are more suitable for local moisturizing and sustained release in fresh-cut fruits, climacteric fruits such as bananas, and small edible fungi. In the future, combining the humidity-regulating ability of hydrogels with the cushioning and liquid-absorbing capacities of aerogels may enable the development of composite gel protective pads better suited to different types of fruits, vegetables, and edible fungi.

## 7. Challenges and Perspectives

### 7.1. Gap Between Model Systems and Real Food Systems

At present, many gel-based absorbent pads are still mainly evaluated in water, buffer solutions, or simulated juices for their water absorption, release, and response performances. However, real food systems are far more complex than model systems. Meat exudates contain proteins, lipids, salts, enzymes, and microbial metabolites, while fruit juices contain sugars, organic acids, pigments, and cell debris. These components may affect gel swelling, pore wetting, active compound release, and color responses. Therefore, high water absorption capacity or ideal release profiles obtained in model systems alone cannot fully demonstrate actual packaging performance. Future studies should more frequently employ real meat, aquatic products, and fruits for validation, and establish correlations between material properties and changes in food quality.

### 7.2. Balance Among Water Absorption Capacity, Liquid Retention Capacity, and Food Quality

For absorbent pads, higher water absorption capacity is not always better. For meat and aquatic products, insufficient liquid absorption can lead to exudate accumulation and microbial proliferation, whereas excessive liquid absorption may also alter the moisture state of the food surface, affecting color, texture, and consumer acceptance. For fruits, absorbent pads need to absorb condensed water and juice released from damaged tissues while avoiding aggravated water loss and wilting. Therefore, the evaluation of gel-based absorbent pads should not focus solely on swelling ratio or water absorption capacity, but should also consider liquid uptake rate, liquid retention under pressure, re-exudation, wet-state strength, and food quality maintenance. In the future, Janus structures, multilayer structures, and gradient pore structures may be used to achieve unidirectional liquid transport and internal liquid storage, thereby reducing liquid backflow while minimizing adverse effects on the food surface.

### 7.3. Difficulty in Precisely Controlling the Timing and Dosage of Active Release

Active preservation absorbent pads are often loaded with essential oils, polyphenols, antibacterial agents, antioxidants, or nano-active components, but the timing and dosage of release remain difficult to precisely control. If burst release is too strong at the early stage of storage, active compounds may be consumed prematurely and may also cause odor, migration, or sensory issues. If release is insufficient, it may be difficult to inhibit microbial proliferation and oxidative deterioration during the middle and later stages of storage. An ideal system should achieve on-demand release in response to exudate accumulation, pH changes, increased enzyme activity, or the generation of microbial metabolites. Future studies should correlate release profiles with real quality indicators such as TVC, TVB-N, thiobarbituric acid reactive substances (TBARS), pH, mold counts, or decay rate, rather than merely demonstrating releasability in vitro without clarifying whether the release behavior truly matches the food spoilage process.

### 7.4. Industrial-Scale Preparation and Cost Issues

Hydrogel and aerogel absorbent pads have shown promising laboratory performance, but their industrial application still faces challenges related to cost, stability, and scalability. Hydrogels have high water content and may encounter problems such as storage instability, microbial contamination, and increased transportation costs. Although aerogels have advantages such as high porosity and rapid liquid uptake, freeze-drying or supercritical drying is costly, and some aerogels suffer from brittleness and batch-to-batch stability issues. Future efforts should focus on developing low-cost, continuous, and scalable fabrication processes, while strengthening evaluations of food-contact safety, active compound migration, nanomaterial risks, and biodegradability. Only by balancing performance, cost, safety, and processing feasibility can gel-based absorbent pads truly enter practical food packaging applications.

## 8. Conclusions

Hydrogels and aerogels provide an important basis for the functional design of absorbent pads in food packaging. Despite their shared three-dimensional polymer networks, these two gel systems differ in their structural features, including hydration state and pore architecture, which in turn dictate distinct functional behaviors in liquid uptake and active compound release. Hydrogels mainly rely on hydrophilic network swelling and liquid confinement, making them suitable for flexible liquid retention, local humidity regulation, and responsive indication. Aerogels, by contrast, depend on dry porous skeletons and capillary action to achieve rapid liquid uptake, liquid storage, cushioning protection, and efficient loading, making them more suitable for exudate management and sustained-release preservation. Their water absorption, liquid retention, release, and indication functions can be further improved through crosslinking regulation, multilayer structures, Janus structures, gradient pore structures, and micro/nano-reinforced architectures. In practical applications, meat and aquatic products place greater emphasis on exudate management, active preservation, and freshness monitoring, whereas fruits, vegetables, and edible fungi require cushioning protection, humidity/juice regulation, and antibacterial/antifungal functions. Future studies should strengthen real food validation, food-contact safety evaluation, precise release control, and scalable fabrication to promote the practical application of gel-based absorbent pads in sustainable food packaging. Beyond these efforts, emerging approaches such as AI-driven material design, 3D-printed gel architectures with precisely controlled pore structures, and fully biodegradable gel systems offer promising avenues for further innovation.

## Figures and Tables

**Table 1:** Representative hydrogel- and aerogel-based absorbent pads for meat and aquatic product packaging.

Matrix Composition	Material Type	Food Sample	Water Absorption/Swelling Data	Key Application Data	Ref.
Commercial absorbent pad	Non-gel absorbent pad	Chicken breast fillets	Not reported	Drip loss was 2.72% after 20 d; pad number explained 88.7% of drip-loss variation	[71]
PBAT/TPS film	Non-gel absorbent pad	Fresh pork	WAC: 56.9%	Day 8 TPC: 7.36 log CFU/g; TVB-N limit was delayed from day 4 to day 6	[72]
Sodium alginate	Hydrogel	Chilled pork	Swelling ratio: 3286.83%	Day 8 TVB-N < 0.15 mg/g; shelf life was extended by 2 d	[28]
Carboxymethyl cellulose/bacterial cellulose/citric acid	Aerogel	Fresh beef	WAC: ~834.1%	Day 7 TVC: 5.05 log CFU/g; Ag migration: 20.2 μg/kg	[68]
Cellulose nanofibers/PVA	Aerogel	Fresh pork	Porosity: 98.07%	Cinnamaldehyde release increased from 10.3% to 68.4%; shelf life was extended by ~4 d	[34]
Sodium alginate/PVA	Aerogel	Salmon	WAC: 2452.31%	Salmon shelf life was extended from 8 to 12 d; complete soil degradation occurred within 21 d	[73]
Sodium alginate/potato starch	Aerogel	Beef	WAC: 2673%; pore size: 167 μm	Day 9 TVB-N: 22.84 mg/100 g; TVC: 5.96 log CFU/g; color changed from purple to blue	[74]
Agarose	Hydrogel	Pork and fish	Pore size: 7703.36 nm; porosity: 91.45%	TVB-N gas LOD: 0.05 mg/dm^3^; response time: 8 min	[75]

Notes: WAC, water absorption capacity; PBAT, polybutylene adipate terephthalate; TPS, thermoplastic starch; PVA, polyvinyl alcohol; TPC, total plate count; TVB-N, total volatile basic nitrogen; TVC, total viable count; Ag, silver; LOD, limit of detection; CFU, colony-forming unit.

**Table 2:** Sample properties and application performance of gel pads in fruit, and vegetable packaging.

Matrix Composition	Material Type	Food Sample	Sample Properties	Key Application Data	Ref.
Chitosan/PVA; chitosan/montmorillonite	Aerogel	Wax apple	Low density; good cyclic stability in the 5% MMT group; relatively uniform pore structure in CM-Cu10	Decay rate decreased by 66%; shelf life extended by 4 d	[80]
Chitosan/montmorillonite/nanocellulose	Aerogel	Blueberry	Density of 0.04–0.06 g/cm^3^; water absorption of approximately 20%; impact-resistant behavior	Antibacterial rate approached 100%; storage period extended by 3 d	[81]
Gelatin/dialdehyde holocellulose nanofibers	Aerogel	Strawberry	Water adsorption of 44.31 g; shrinkage rate of 3.49%	Weight loss reduced by 42.21% on day 5; TVC decreased to 3.26 log CFU/g	[82]
Bamboo shoot shell cellulose/dialdehyde cellulose/chitosan	Aerogel	Strawberry	Moisture adsorption/desorption of 22.57%/15.19%; compressive strength of 264.68 kPa	Shelf life extended from 5 d to approximately 9 d; antibacterial activity maintained for 21 d	[83]
Carboxymethyl cellulose/PVA	Hydrogel	Banana	Transparent; good tensile performance; complete degradation within 50 d	Weight loss decreased from 8.83% to 1.37% after 3 d	[84]

Notes: PVA, polyvinyl alcohol; MMT, montmorillonite; CM-Cu, chitosan/montmorillonite/copper nanoparticle-loaded antibacterial fiber aerogel; TVC, total viable count; CFU, colony-forming unit.

## Data Availability

No new data were generated or analyzed during the preparation of this review article.
